# An Ecological Profile of *Hydropsyche alternans* (Trichoptera: Hydropsychidae) in Lake Superior, the Last Stronghold of a Once-Dominant Great Lakes Surf Zone Caddisfly

**DOI:** 10.3390/insects13070659

**Published:** 2022-07-21

**Authors:** Sam Miess, Alissa Chrisekos, Mac Strand

**Affiliations:** Department of Biology, Northern Michigan University, Marquette, MI 49855, USA; smiess@nmu.edu (S.M.); achrisek@nmu.edu (A.C.)

**Keywords:** Hydropsychidae, Lake Superior, surf zone, trophic ecology, autecology

## Abstract

**Simple Summary:**

Prior to the invasion and spread of *Dreissena* mussels in the late 1980s, the nearshore waters of the Laurentian Great Lakes were home to diverse assemblages of native aquatic insects, including the net-spinning caddisfly *Hydropsyche alternans,* which occurred in rocky surf-exposed habitat throughout the system from Lake Superior downstream to Lake Ontario. These surf zone caddisflies are still abundant in the largely *Dreissena*-free waters of Lake Superior where the present study was conducted. They have not been reported in the lakes below Lake Superior for decades, and are presumed to have been extirpated from *Dreissena*-infested habitats. The ecological profile presented here documents the life history of *H. alternans* in Lake Superior, and reveals details of its feeding biology that shed light on the roles that surf zone net-spinners play in the native nearshore food webs of the Great Lakes. The *H. alternans* life history in Lake Superior begins in mid-summer shortly after oviposition, which features females swimming to surf-exposed lake bottom substrates. Larval development takes approximately 10 months, but most of it occurs during their first 100 days. Adult emergence is broadly synchronous, with large numbers present from the summer solstice through mid-July. Gut content analyses showed that larvae opportunistically feed on algal, animal, and detrital material of aquatic and terrestrial origin. δ^13^C and δ^15^N stable isotope data indicate that they function as omnivores that link coastal, nearshore, and pelagic food webs. These energetic links, and the very existence of surf zone insect assemblages in the Great Lakes, depend on the *Dreissena* populations in Lake Superior remaining relatively small and isolated.

**Abstract:**

We studied the life history, diet, and trophic ecology of *Hydropsyche alternans* in four rocky sites located along the south-central coast of Lake Superior. The *H. alternans* life history and broad trophic niche space were similar to those of its riverine relatives. Quantitative sampling over the course of one ice-free season revealed that most individuals lived univoltine life histories that featured early to mid-summer mating, and oviposition and rapid growth and development through summer into fall. Most individuals overwintered as ultimate or penultimate larval instars. Pupation followed ice-out in the spring. Gut content sampling and δ^13^C and δ^15^N stable isotope analyses indicated that the typical larval diet is a mix of benthic, pelagic, and terrestrial food resources, including diatoms, small arthropods, sloughed periphyton, and in one site, fugal hyphae apparently of foredune origin. As a suspension-feeding omnivore that relies on waves and currents to deliver food to its nets, *H. alternans* larvae form energetic links between coastal, nearshore, and offshore food webs. These connections have been lost throughout the lower Laurentian Great Lakes as a consequence of the invasion and spread of *Dreissena* mussels.

## 1. Introduction

Net-spinning caddisflies (Trichoptera: Hydropsychidae) are among the most well-known aquatic insects. Larval hydropsychids filter food particles from flowing water using silken mesh nets rigged to retreats made of silk and bits of mineral and plant material. These little “fishing shacks” are common features of stream bottoms worldwide. Hydropsychids also inhabit the rocky, wave-swept shorelines of large lakes alongside other familiar stream insects such as flathead mayflies (Ephemeroptera: Heptageniidae), perlid stoneflies (Plecoptera: Perlidae), riffle beetles (Coleoptera: Elmidae), and *Antocha* crane flies (Diptera: Limoniidae) [1,2,3].

The flows that impart a riverine nature to the nearshore waters of large lakes are produced by wave energy surging up beaches and running back out, forming longshore currents during prolonged wind events [4]. In very large lakes, of which there are 253, globally exceeding 500 km^2^ in surface area [5], wind energy can produce circulating lake-wide currents and strong surf along exposed shores [4,6,7,8]. Rocky surf zone habitats in these lakes host a wide variety of insects, including larval Hydropsychidae, which typically occur in lean-to shelters built with silk and gravel within cracks and crevices in bedrock and boulders [1].

Waves and currents deliver suspended food particles to surf zone hydropsychid nets. By limiting the accumulation of fine sediments [1,2], wave energy also promotes the establishment of benthic diatoms, a staple food source of many flowing water insects [9], including hydropsychids, which capture sloughed cells in their nets and graze directly on epilithic forms [10]. Large lake net-spinners also presumably enjoy periodic deliveries of planktonic food drawn into the surf zone with upwelling pelagic water following strong offshore winds. Long-duration winds and rapidly travelling storms can also feed offshore energy to the surf zone by setting in motion internal gravity waves called seiches, which travel throughout lakes, mixing nearshore and offshore waters and connecting coastal and pelagic food webs [11].

Like many aquatic insects, larval Hydropsychidae are omnivorous feeders [12]. Gut content analyses have shown hydropsychid diets to be a mix of small animals and algae, with distinct changes coinciding with developmental stage and season [10,13,14,15,16,17]. Lake flows that deliver pelagic food items to the surf zone also transport nearshore benthic energy in the form of sloughed periphyton and relocating invertebrates, as well as terrestrial subsidies washed in from shore [18]. Therefore, surf zone hydropsychids likely have access to a very broad range of food resources, including epilithic and planktonic algae, benthic and planktonic invertebrates, and terrestrial plants and animals. These food sources can be gleaned from nets with labral bristles, grazed from substrates, or collected and macerated with their formidable mandibles [10,15,19].

Stable isotope studies have shown that larval Hydropsychidae tend to fall in the middle of available energy sources within a system, and that their trophic position ranges from primary consumer (trophic level 2) to predators of small invertebrates (trophic level 3) [3,17,20,21,22,23,24]. Past observations from the south-central shore of Lake Superior indicate that surf zone net-spinners occupy a particularly broad trophic space within this system, incorporating benthic and planktonic energy as primary and secondary consumers [3]. It is not known whether this apparently broad trophic range resulted from highly variable diets among all individuals within habitats, or from life history factors, e.g., increasing carnivory with increasing age, or site-specific factors, e.g., differing exposure to currents carrying pelagic resources.

Prior to the invasion of *Dreissena* mussels, hydropsychids were common in the nearshore waters of all five Laurentian Great Lakes [1]. As populations of zebra mussels (*Dreissena polymorpha*) and quagga mussels (*Dreissena bugensis*) expanded throughout Lakes Michigan, Huron, Erie, and Ontario over the past three decades [25,26], with widely reported average abundances of thousands of mussels per square meter [27,28], surf zone caddisflies, along with many other aquatic insects, have nearly disappeared from Great Lakes habitats where *Dreissena* populations are thriving [29,30]. Although far too few studies have been conducted in the nearshore waters of the Great Lakes to know for certain [31], it presently appears as though intact native surf zone insect communities occur only in Lake Superior. Along the boulder-strewn sandstone pavement lake bottom of Lake Superior’s south-central shore, the most lacustrine hydropsychid and the star of this paper, *Hydropsyche alternans* (Walsh), still flourishes in the last stronghold of the Great Lakes surf zone insects.

### Habitat and Historical Biogeography

While describing a southern Lake Michigan population of the species he knew as *Hydropsyche recurvata* (see Supplementary Materials S1:Taxonomic History), legendary trichopterist Herbert H. Ross [32] was first to write about the dual identity of *H. alternans*, which seems to be equally at home in cold, swift rivers and the wave-swept littoral zones of large lakes. Barton and Hynes [1] suggested that this high degree of habitat flexibility may have been key to the divergence of this lotic lineage into lacustrine habitats. Observations by Schmude and Hilsenhoff [33] indicate that this flexibility also extends to the dynamic chemical environments of some lacustrine habitats. Although they determined that the species referred to as *Ceratopsyche alternans* (see Supplementary Materials S1: Taxonomic History) was more sensitive to organic enrichment than other Wisconsin (USA) *Hydropsyche* species, a subpopulation of *H. alternans* in a lacustrine zone (Lake Butte des Morts) of the Fox River, the largest tributary of Lake Michigan, persisted in shallow water lake margins despite massive cyanobacterial blooms during summer months and the dramatic water quality changes that followed. This apparent tolerance of widely fluctuating lacustrine water quality was aptly deemed “remarkable” by the authors.

The present distribution of *H. alternans* suggests a strong influence of Pleistocene glacial–interglacial cycles in shaping its broad niche space [34]. Williams and Eyles [35] surveyed the caddisfly fauna assembled from sclerotized exoskeleton fragments collected from an 80,000-year-old glacial deposit just north of the modern Lake Ontario shoreline. *Hydropsyche alternans* dominated the insect assemblage of the large lake that formed there during the final interglacial (warm) period before the advance of the Laurentide Ice Sheet that ultimately carved out the modern Great Lakes. Eleven interglacials, which are separated by long periods of cold temperatures and advancing glaciers, have occurred over the past 800,000 years [36]. Although the fossil record of aquatic insects from Pleistocene interglacial streams and lakes is understandably scarce, it is easy to imagine that the *Hydropsyche alternans* range expanded from unglaciated refugia streams into large glacial scour lakes formed during interglacial periods, as it has during the present (Holocene) interglacial, which began approximately 11,700 years ago.

**Figure 1:**
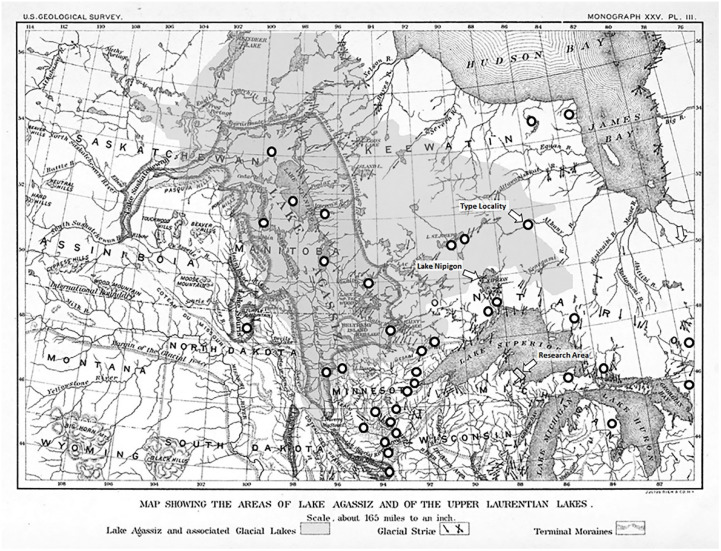
Late Pleistocene glacial geology map showing Lake Agassi and the approximate locations of published collection records from the central part of the range of *Hydropsyche alternans.* Base map, Plate III from [45]; overlay (gray shading): Lake Agassiz total geographic extent follows Figure 1 from [46]. Collection records from [32,42,47,48,49,50]. Additional distribution information from within the boundaries of this map not shown: multiple locations in Lakes Erie, Huron, and Superior [1,3] multiple streams in northern and central Wisconsin [33], multiple streams in northern Michigan [51].

Published collection records from its modern range indicate that the ancestors of Great Lakes *H. alternans* may also have occurred in glacial meltwater lakes, including the glacial Lake Agassiz, the immense proglacial lake that extended up to 500 km outward from the Laurentide Ice Sheet (Figure 1). Lake Agassiz, which had a maximum volume 1.5 times greater than the total volume of the modern Great Lakes [37], was inhabited by the ancestors of many of the species that populated the newly forming Great Lakes. Over its brief 4000-year existence at the end of the Pleistocene, Lake Agassiz had outflow connections to the Arctic Ocean, the Gulf of Mexico, Hudson Bay (North Atlantic), and the North Atlantic, through the Great Lakes and the St. Lawrence River [38]. The Great Lakes connection occurred approximately 10,400 years ago, when water carrying suspended organisms from Lake Agassiz drained into the basin that now holds Lake Nipigon, which expanded and overflowed into the Lake Superior basin [39,40]. Inter-system comparisons by Patalas [41] focused on modern Lake Winnipeg and other lakes within the ancestral Lake Agassiz basin, indicated that almost all of the zooplankton populations in the Great Lakes were founded via emigration from Lake Agassiz. The origins of the aquatic insect fauna of the Great Lakes likely consist of a mix of aerial colonization from distant sources and within-basin aerial and fluvial movement. Neave’s 1934 [42] report of *H. alternans* being common and widespread in Lake Winnipeg, an extant remnant of Lake Agassiz, along with the distribution information presented in (Figure 1), suggests that the Lake Superior population of the present study was founded by colonists from lacustrine or riverine populations from the Lake Agassiz basin. The modern *H. alternans* range extends northwest to waters flowing to the Gulf of Alaska and east through the former basin of Glacial Lake Chippewa [43] and the St. Lawrence River to Lake Champlain [44]. This transcontinental distribution indicates that the recently extirpated populations in the lower Great Lakes may have emigrated from eastern refugia, as these oldest Great Lakes basins became habitable at the end of the Pleistocene.

This paper documents the autecology of *H. alternans* in one of its last Great Lakes strongholds along a 20 km stretch of coastline on Michigan’s Upper Peninsula. Our approach combined introduced substrate sampling, gut content analysis, and δ^13^C and δ^15^N stable isotope analyses to reveal details of the life history, diet, and trophic niche space of this remarkable caddisfly. 

## 2. Materials and Methods

### 2.1. Research Area

**Figure 2:**
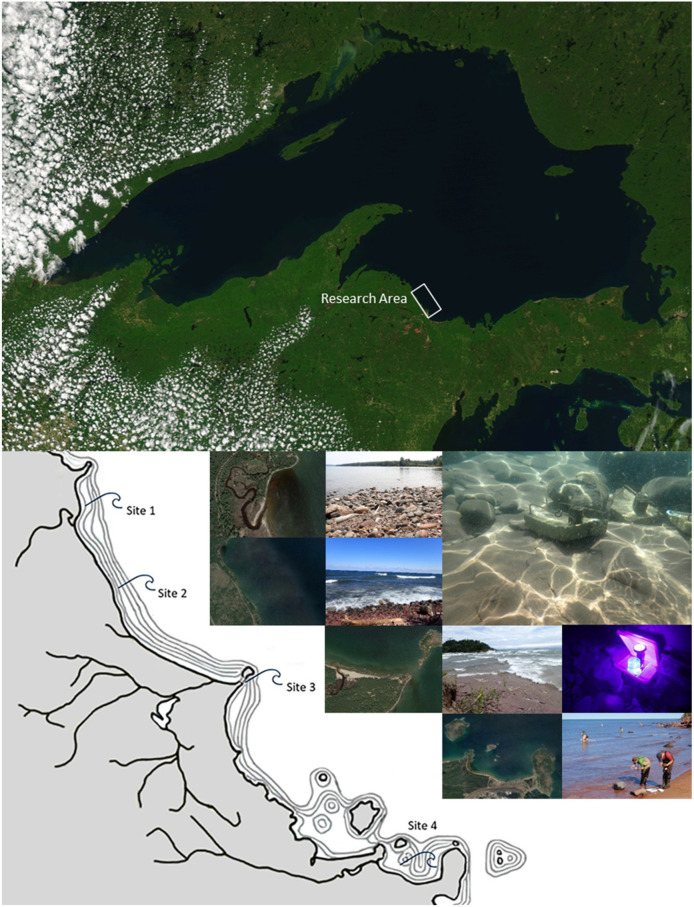
Research area, site locations, and caddisfly sampling equipment. Images clockwise from top: Lake Superior MODIS image showing location of research area [52]; deployed samplers underwater showing sandstone and boulder lake bottom habitat; UV light adult caddisfly sampler; research area showing location, satellite images [53], and photographs of the four sites. Detailed site descriptions are presented in Supplementary Materials S1: Site Descriptions.

The research presented here was conducted in four sites in the vicinity of Marquette, Michigan, USA (Figure 2). Detailed site descriptions are presented in Supplementary Materials S1: Site Descriptions.

Hydropsychidae habitat quality is largely determined by a combination of the availability of solid substrates suitable for net and retreat construction, and moving water to deliver food. Most retreat sites within our research area (Figure 2) are found along exposed slabs of Jacobsville sandstone and among boulders comprising sandstone and a wide variety of igneous and metamorphic rock fractured from local strata or deposited by Pleistocene glaciers.

All four sites, which are numbered 1–4 north to south, are protected from westerly winds and experience daily lake breezes during much of the open-water season. These onshore winds, which occur when heat on land produces updrafts that draw cool lake air shoreward, are a common feature of lakes large enough to support hydropsychids [2]. Evidence of lake breezes can be seen in the cloud patterns over Lake Superior on the satellite photo in Figure 2. All sites also experience periodic upwelling events and seiches. The effects of these mixing events at the site-specific level are not known, but due to the close proximity of our sites to each other and the generally similar coastal aspect, we expect that large-scale upwellings and seiches affect all of the sites to varying degrees.

The three northernmost sites (Sites 1–3) are located along a 7 km stretch of northeast facing coast. Granitic headlands to the north (Thoney Point) and south (Little Presque Isle) exert considerable influence on the surf and current exposure. All of these sites periodically receive strong surf from long-duration gale-force (≥40 km per hour) north, northwest, northeast, and easterly winds. The larval retreat sites in Site 4 occur along the west side of a granitic headland that forms an embayment which captures wind swells arriving from the northwest, north, and northeast.

### 2.2. Life History Analysis

Larvae used for life history analyses were collected with multiple-plate samplers [54] in an 8-plate, 0.1 m^2^ configuration [55]. Samplers were attached with 0.3 cm flexible steel wire to 0.6 steel rebar handles embedded in 37 × 28 × 7.5 cm poured concrete substrates. Substrates were placed in approximately 1.5 m deep water (Figure 2), approximately 8 m from shore, at Sites 1 and 2. Seven samplers were deployed at each site during each sampling period. Samplers were deployed for 4 sequential, approximately 28-day sampling periods over June–September 2020. Samplers were removed from the lake by a diver who bagged each in a 225-micron mesh bag (17 cm round opening, 40 cm long) while underwater, taking care to minimize handling until the sampler was safely in the bag. Samplers were disassembled on shore. Plates, invertebrates, and associated debris were placed in plastic boxes, covered in 95% ETOH, and transported to the lab. Macroscopic animals were picked from samples under 10–40× magnification. Larval caddisfly head measurements were taken using an ACU-RITE linear encoder measuring system. Larval identification followed [49]; adult identification followed [32,56].

#### 2.2.1. Larval Instar Determination

Larval Hydropsychidae pass through five instars prior to pupation. As they grow, the head capsule width of successive instars increases in a consistent geometric progression [57], typically by a factor of approximately 1.5 [58]. By collecting a relatively large number (1919) of *H. alternans* larvae over the course of a full growing season and by accurately measuring the widths of their heads, we were able to reliably differentiate among larval instars. Additionally, identification of 5th instar Hydropsychidae by head capsule width can be verified by the presence of bud-like pupal gills on the sides of the abdomen [59].

#### 2.2.2. Adult Flight Period

The approximate beginning, peak, and end of the adult flight period were determined with evening collections made with a portable light trap equipped with a 395 nm LED flashlight (Figure 2) six times during the summer of 2020 at Sites 1 and 2. Sample dates were 18 June, 28 June, 13 July, 17 July, 23 July, and 14 August. A small number of *H. alternans* adults from each sample were identified using male genitalia characteristics, which allowed us to establish adult presence. Peak abundance was determined when the sampling jars containing 95% ethanol were overflowing with thousands of specimens after approximately 6 h of sampling.

#### 2.2.3. Adult Leg Morphology

Most female Hydropsychidae oviposit underwater and some species are known to swim to substrates using flattened mesothoracic legs, a behavior that would presumably be adaptive in Lake Superior where suitable habitat extends from shore for hundreds of meters in many locations. To assess the potential for female swimming and deep oviposition in *H. alternans*, we calculated the length:width ratio of the first mesothoracic tarsal segments of 10 females and 10 males with an ACU-RITE linear encoder measuring system. Although not definitive evidence of swimming behavior, significant sexual dimorphism in this character has been linked to swimming and deep oviposition in hydropsychid species [60,61].

### 2.3. Gut Content Analyses

Gut content analyses were performed on 39 larvae from Site 4 (9 fourth instar and 13 fifth instars) collected on 6 October 2017 and 17 larvae from Site 3 (4 fourth instars, 12 fifth instars, and 1 unknown instar due to lost head) collected on 16 October 2017. Gut content samples were processed and prepared using Cummins’ standard protocols [62]. Foregut and midgut contents were slide mounted with Euparal medium. Slides were analyzed under 40–1000× magnification. Large food items were highly fragmented by the chewing and grinding actions of the mandibles and proventriculus. This generally limited the taxonomic precision of our identifications. Identifiable items were categorized as algae, animal, fungus, sediment, or amorphous matter.

### 2.4. Stable Isotope Analyses

After removing the foregut and midgut for gut content analyses, specimens were processed for stable isotope analysis by removing the hindgut and the head, which was retained as a voucher specimen. The rest of the specimen was then dried at 55 °C and sent to the Alaska Isotope Facility for sample preparation and δ^13^C and δ^15^N stable isotope analyses.

Specimens from Sites 1 and 2 were processed using a modified protocol. While removing the midgut and hindgut, which is standard practice, particularly for predators or omnivores [21], we noticed considerable variation in the silk gland fullness among individuals. Because silk glands are full of dense granules of silk precursor materials and a gel-like protein polymer that is extruded as silk [63], we surmised that silk glands are likely to be isotopically distinct from the rest of the body, which could lead to the misinterpretation of isotope data from individuals with differing gland fullness at the time of collection. To test this supposition, glands were removed and analyzed separately. The bodies of three 4th instar larvae, and three 5th instar larvae collected in July 2020 from Sites 1 and 2, and their silk glands were analyzed. Silk glands were pooled by instar and site. A technical error made it impossible to assign larval bodies to either of the two 4th instar silk gland samples from Site 2. One of these samples certainly contained the silk glands from the three 4th instar *H. alternans* larvae included in the body tissue analysis. The other sample contained silk glands from two 4th instar *H. alternans* and one similarly sized *Cheumatopsyche* larva. Data from both samples, which were isotopically similar and thus of general value in assessing the relationship between 4th instar *H. alternans* body and silk gland isotopic compositions, are included in the results. 

Although the focus of the present study was on larval trophic ecology, three adult males and three adult females from Sites 1 and 2 were analyzed in order to characterize differences in resource allocation that occur as male and female reproductive tissues differentiate. These samples will also serve as the baseline data for future investigations of energy flow from the surf zone to the swash zone, and nearshore terrestrial food webs in the Lake Superior basin.

### 2.5. Data Analyses

Unpaired (independent samples), two-tailed t-tests, with an a priori significance level of *p* ≤ 0.05 were used to evaluate the observed differences in adult vs. larval body δ^13^C and δ^15^N, adult male vs. female body δ^13^C and δ^15^N, and sexual dimorphism in adult legs. Single factor ANOVA models were used to test the effects of larval instar and site on isotopic ratios, and the effects of instar and site on larval silk gland and body isotopic ratios. Post hoc Tukey–Kramer tests were used to test for pairwise differences among treatment means. All data analyzed met the assumptions of independence and normality (Kolmogorov–Smirnov tests). All isotope data met the assumption of homogeneity of variance (Levine’s tests). Adult leg data were log transformed to correct for heteroskedasticity.

## 3. Results

### 3.1. Life History Analysis

UV light sampling detected a small number of adults on 18 June, establishing the approximate beginning of the 2020 flight period. Adults were abundant from late June through mid-July, tapering off dramatically by early August. During the daylight hours, adults nervously rested on lakeside vegetation and other shaded habitats. Small, loose swarms, approximately 2 to 5 m above ground along the forest–beach edge were generally present throughout the day. At dusk, adults appeared en masse along the shoreline. Pre-mating behavior featured a dizzying blend of low swarming flights, frenetic scrambling over, under, and around lakeshore rocks, and excited nuzzling by males alongside of females emitting pheromonal signals from paired abdominal glands [64].

**Figure 3:**
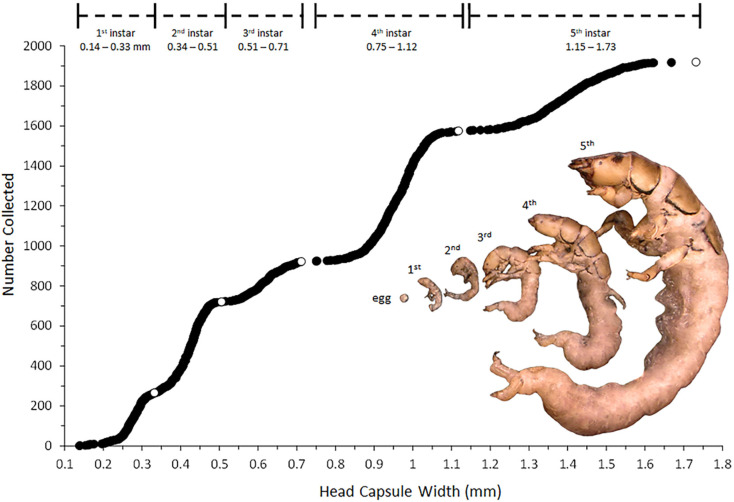
Larval instar determinations using head capsule width distribution. Each circle marker represents an individual larva (n = 1919). Open circles represent the widest head capsule observed for each instar. The inset photo shows the relative sizes of preserved specimens of each life stage.

**Figure 4:**
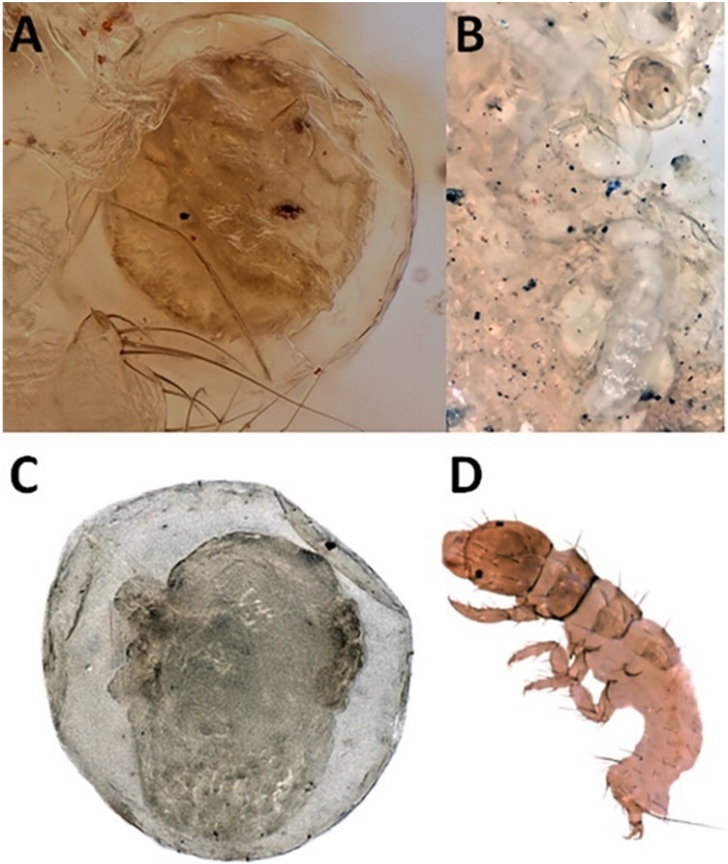
Early life stages of *Hydropsyche alternans*. (**A**) Egg, early embryo stage; (**B**) egg mass with developing eggs and newly hatched, unsclerotized, 1st instar larvae, which remain in the egg mass for a short time before emerging; sibling cannibalism is a major source of mortality during this period [65]; (**C**) egg, late stage embryo, its head is at the top, developing legs are visible along its sides; (**D**) fully sclerotized 1st instar larva.

**Figure 5:**
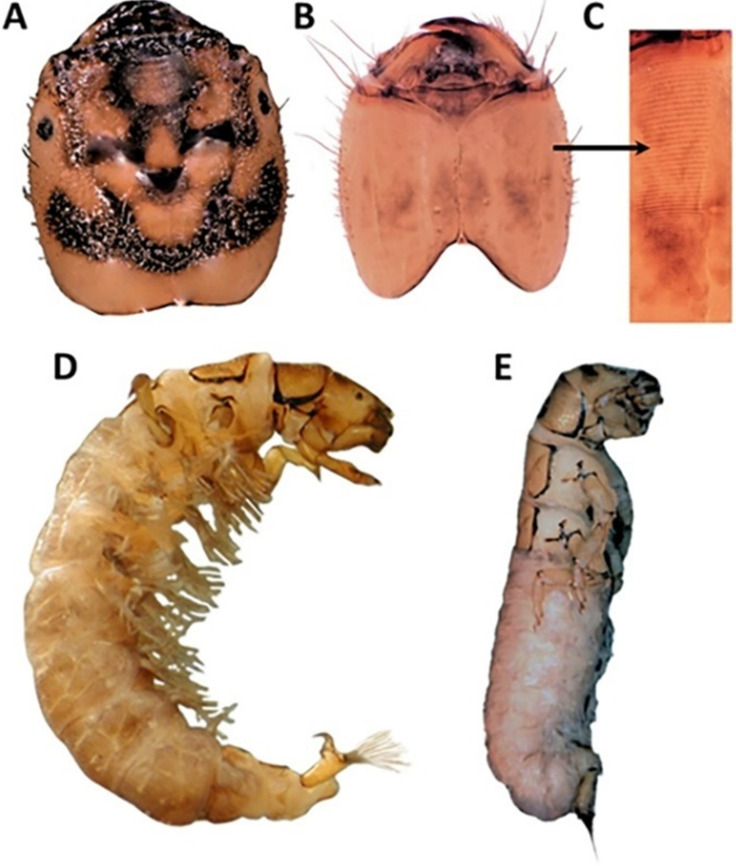
Late-stage larval morphology of *Hydropsyche alternans*. (**A**) Fifth instar head, dorsal view showing quadrate head shape and markings; (**B**) 5th instar head, ventral view showing large mandibles, sensory sensilla and setae, and stridulatory files; (**C**) high contrast image of left file; (**D**) 5th instar larva; (**E**) pre-pupa.

**Figure 6:**
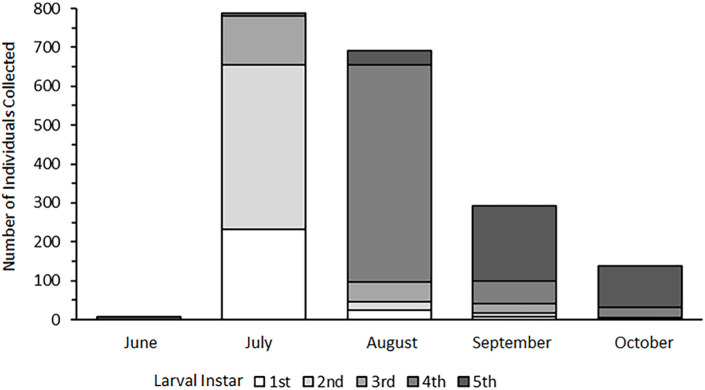
Stage-specific abundance data from introduced substrate sampling in Sites 1 and 2 during the open water season of 2020.

Head width data, instar determination cutoff points, and the relative sizes of egg and larval life stages are shown in Figure 3. The images of the early and late developmental stages are presented in Figure 4 and Figure 5, respectively. As can be seen in Figure 3, a variation in body size and general body shape can also be used to inform instar determinations. Stage-specific life history data are presented in Figure 6. The first *H. alternans* eggs were observed on larval samplers in late July, although adult collections showing oviposition were likely occurring at least a month earlier. This is further supported by the presence of large numbers of 2nd and 3rd instar larvae in the late July samples. Larval development appeared to be largely completed by fall, with most individuals overwintering as 5th instars. However, the presence of some 4th instar larvae in early winter samples indicates a degree of developmental flexibility at the end of a larva’s first open-water season.

### 3.2. Adult Morphology

**Figure 7:**
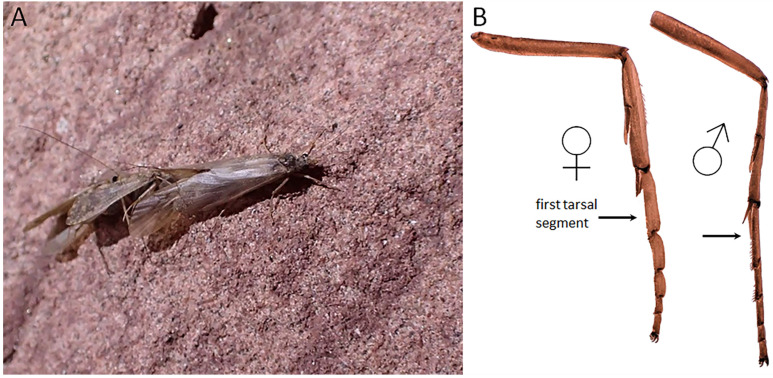
*Hydropsyche alternans* adult characteristics. (**A**) Mating behavior, a female (center right) being examined by a male (under her wing); (**B**) female and male mesothoracic legs showing sexual dimorphism, widened female middle legs are a common feature hydropsychid females that dive and swim to oviposition sites.

The first tarsal segments of the 10 females measured were much wider relative to their length, than were those of the 10 males measured (average L:W ± 1 SE, t(df) = 3.54 ± 0.08 for females, 6.99 ± 0.22 for males, t(18) = 18.32, *p* < 0.00001) (Figure 7). Our samplers were approximately 1.5 m deep and 8 m offshore. Thus, the presence of Hydropsychidae egg masses on our samplers suggests that surf zone hydropsychid females are capable of diving and swimming to oviposition substrates. First instar larvae can be seen eclosing from eggs laid on one of the samplers (Figure 4B).

### 3.3. Gut Content Analyses

**Figure 8:**
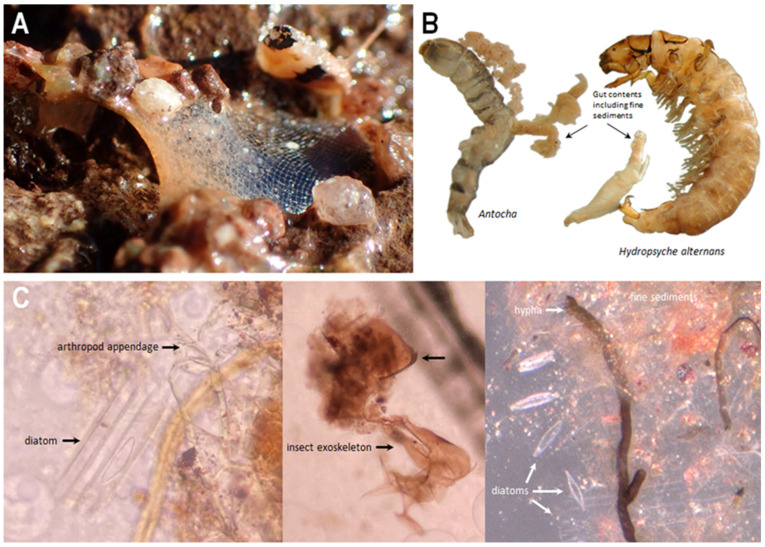
*Hydropsyche alternans* feeding and diet, (**A**) silken net filled with fine particles; (**B**) bodies and partially removed gut contents of *Antocha* (Diptera: Limoniidae) and *H. alternans* showing fine sediment in guts; (**C**) micrographs of *H. alternans* gut content items.

Although a very general level of identification of gut content items was employed here, a close look at the partially masticated remains in the guts of a late-instar *H. alternans* collected in October from Sites 3 and 4 indicates that a wide variety of food items were consumed (Figure 8). Algal remains were green algae cell wall fragments and diatom frustules. Animal remains included bits of exoskeleton and chitinous spines from arthropod prey. Fungal remains were pieces of hyphae. The ingested sediments were fine sand particles apparently bound in amorphous organic material, as could be expected if this material was consumed along with epilithic and epipsammic diatoms (Figure 8B,C).

**Figure 9:**
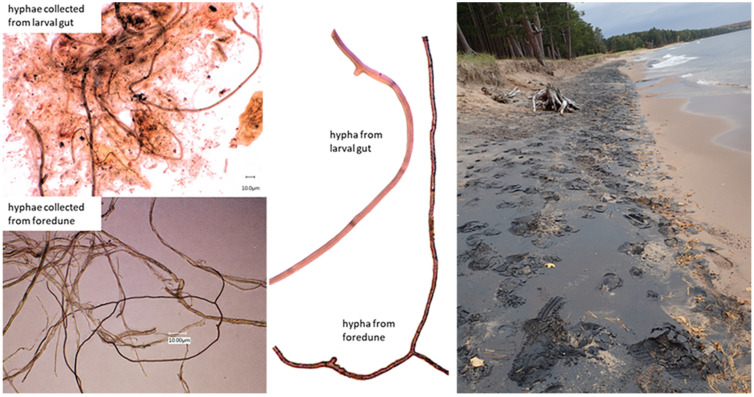
Fungal hyphae from Site 3 larval guts and their likely origin; left and center: side-by-side comparisons of fungal hyphae from the gut contents of a late-instar larval *H. alternans* and hyphae from the eroding face of a beach dune at Site 3; Right: beach at Site 3 facing north; the *H. alternans* habitat from which specimens were collected lies just to the south of this position. Eroding dune faces are visible at the beach–forest transition on the upper right. Dune grass stems, roots, and associated fungal hyphae are concentrated along the swash line on the lower right. The black sediment is magnetite (Fe_3_O_4_) ground from bedrock by Pleistocene glacial ice and deposited at the high-water mark during the storm surge that eroded and carried the dune grass material into the surf zone.

Larvae from both sites (Sites 3 and 4) had broadly similar diets and were clearly omnivorous. Approximately half of the larvae examined had recently consumed arthropod prey, supplementing common diets comprising diatoms, green algae, and detritus (Table 1). There was, however, one interesting difference between the sites: most of the larvae from Site 3 had recently consumed fungal hyphae. The origin of these hyphae is unknown; however, we suspect that they were washed in from the eroding dune front on the shoreline of this site. Hyphae collected from the dune front appear to be very similar to those found in the guts of the caddisflies from this site (Figure 9). To our knowledge, mycophagy was previously unknown in the Hydropsychidae, and is probably limited to specific ecological settings, but it was apparently widespread at this site in mid-October 2017.

### 3.4. Stable Isotope Analyses

δ^13^C and δ^15^N isotope analyses were conducted on the specimens used in the 2017 gut content analysis from Sites 3 and 4, and also on some of the larvae and adults collected from Sites 1 and 2 as part of the 2020 life history analysis. The data from these analyses, which are treated in this section as four separate studies, are presented together in Table 2. The four studies are: (1) Larval Instar Effects—a comparison of 3rd, 4th, and 5th instar larvae from Site 3 that tests for trophic differences over a broad larval size range; (2) Intersite Variation and Gut Content Associations—a comparison of 4th and 5th instar larvae from Sites 3 and 4 that tests for the effects of site and instar, and also allowed us to look for associations between a larva’s gut contents and its isotopic signatures; (3) Silk Glands—a comparison of 4th and 5th instar larvae from Sites 1 and 2 and their silk glands; and (4) Adults—a comparison of adult males and females from Sites 1 and 2.

#### 3.4.1. Larval Instar Effects

**Figure 10:**
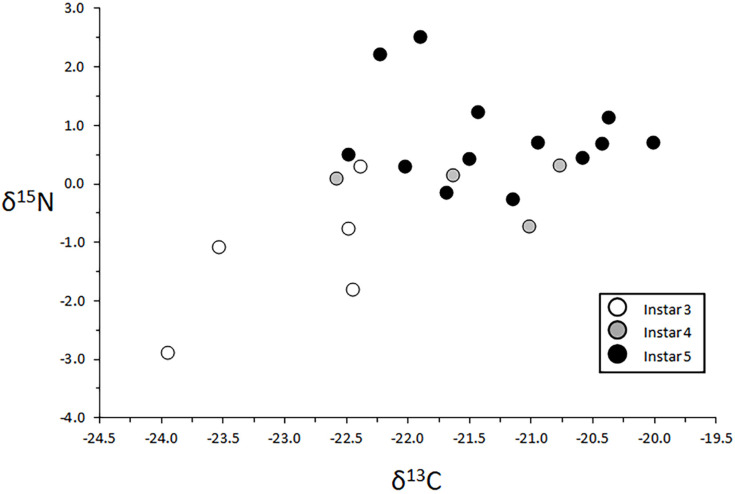
δ^13^C and δ^15^N data for 3rd, 4th, and 5th instar larvae from Site 3.

This study compared 3rd, 4th, and 5th instar larvae from Site 3. An ANOVA model that included δ^13^C data detected significant effects of instar on the principal energy pathway (F = 8.23, *p* = 0.0003). Specifically, 3rd instars were, on average, depleted in ^13^C relative to the 4th and 5th instars, but some overlap with both older instars was apparent as well (Table 2, Figure 10). The corresponding δ^15^N ANOVA model revealed strong effects of instar (F = 10.30, *p* = 0.0009). A post-hoc analysis showed that this pattern was driven by apparent broad trophic separation between 3rd instars and the more ^15^N-enriched 5th instars (Table 3, Figure 10).

#### 3.4.2. Intersite Variation and Gut Content Associations

**Figure 11:**
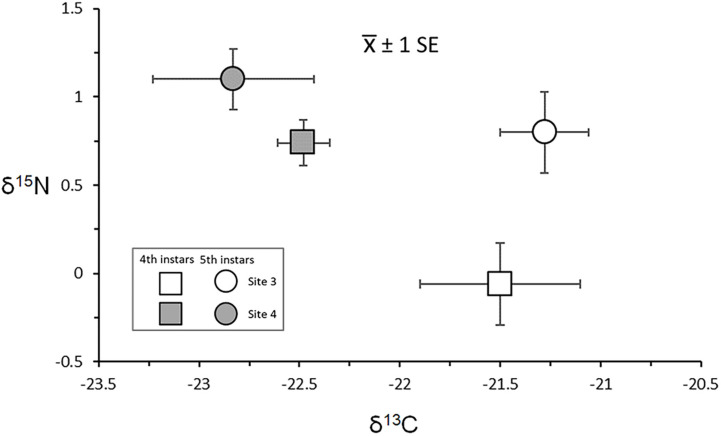
δ^13^C and δ^15^N means and standard errors for 4th and 5th instar larvae from Sites 3 and 4.

This study compared δ^13^C and δ^15^N values from 4th and 5th instar larvae collected from Sites 3 and 4 in October 2017. An ANOVA model that included δ^13^C data detected a significant effect of site (F = 8.2263, *p* = 0.0003) (Figure 11). Post-hoc analysis showed that 4th and 5th instar larvae from Site 3 were enriched in ^13^C relative to Site 4 larvae, and that within sites, 4th and 5th instars had similar δ^13^C signatures (Table 4). The corresponding δ^15^N ANOVA model revealed significant effects of site and instar (F = 3.2407, *p* = 0.0339). Post-hoc analysis showed this effect resulted from apparent trophic separation between the most ^15^N-enriched group, 5th instars from Site 4, and the most depleted group, 4th instars from Site 3 (Table 4, Figure 11).

**Figure 12:**
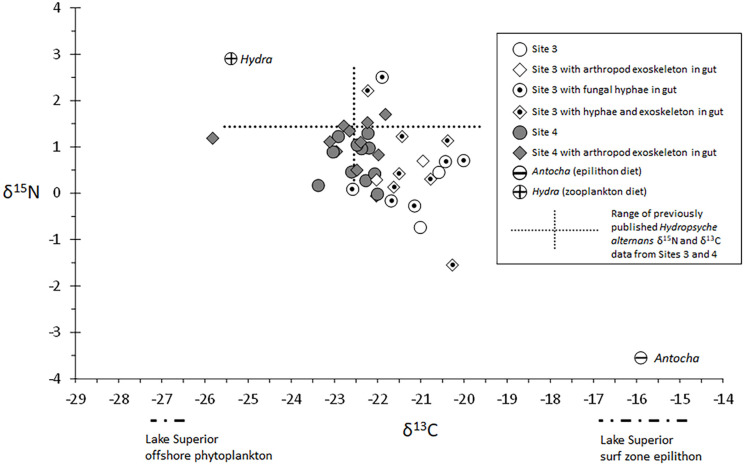
Stable isotope data for 4th and 5th instar *Hydropsyche alternans* from Sites 3 and 4. Positions of *Hydra* (Cnidaria, Hydrozoa, and Hydridae) and *Antocha* (Insecta, Diptera, and Limoniidae) are included to show the relative positions of a trophic level 3 planktivore (*Hydra*) within this food web, and a trophic level 2 benthivore (*Antocha*) that commonly occurs in close association with Great Lakes surf zone Hydropsychidae retreats (Barton and Hynes 1978) [1]. Increasing trophic level is indicated by increasing δ^15^N values. Within this range, more negative δ^13^C values indicate pelagic (planktonic) energy. Less negative δ^13^C values indicate benthic energy. *Antocha, Hydra*, and *Hydropsyche alternans* δ^15^N and δ^13^C data and δ^13^C data for Lake Superior surf zone epilithon are from [3]. Lake Superior offshore phytoplankton δ^13^C data are from [66].

Arthropod exoskeleton fragments were found in approximately 50% of the guts analyzed (Table 1). This recent carnivory had no apparent effect on δ^15^N values (Figure 12), indicating that prey-derived ^15^N may have contributed to all of our observations. Similarly, larvae without fungal hyphae in their guts had δ^15^N and δ^13^C signatures that fell within the range of variation observed in larvae that had recently eaten hyphae (Figure 12), suggesting that if fungal consumption affected their body isotopic compositions, it was a general effect for all Site 3 larvae.

#### 3.4.3. Silk Glands

**Figure 13:**
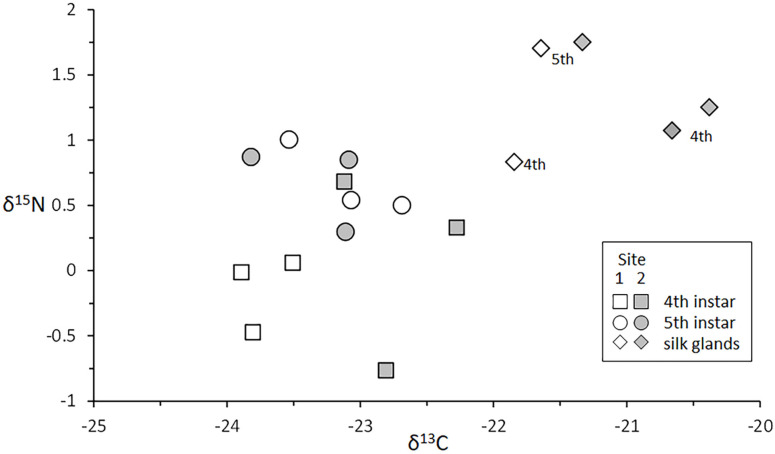
δ^13^C and δ^15^N data for 4th and 5th instar larvae and their silk glands from Sites 1 and 2. Numbers next to silk gland values indicate larval instar associations.

This study compared 4th and 5th instar larval bodies and silk glands collected from Sites 1 and 2, as part of the artificial substrate sampling-based life history analysis (Section 3.1). Results are presented in Figure 13. An ANOVA model that compared larval body mean δ^13^C values indicated little difference between instars or sites (F = 3.68, *p* = 0.0623), although a post-hoc Tukey test detected that 4th instars from Site 1 were slightly depleted in ^13^C relative to 4th instars from Site 2 (Tukey HSD *p* = 0.0480). The corresponding ANOVA model for δ^15^N showed no trophic separation between instars or sites (F = 2.50, *p* = 0.1332).

Larval silk glands were significantly enriched in both ^13^C and ^15^N, relative to bodies (δ^13^C t(15) = 7.18, *p* < 0.00001; δ^15^N t(15) = 3.68, *p* = 0.0022). These results were consistent with contrasts addressing the discrepancy that resulted in two silk gland samples from 4th instars of Site 2 (without silk gland sample 1, t = 6.38, *p* = 000017; without silk gland sample 2, t = 3.52, *p* = 0.0034). Within Site 1, 5th instar larval silk glands were enriched in ^13^C by approximately 9.4% (silk gland δ^13^C vs. mean body δ^13^C = 21.64 vs. −23.10) and contained approximately 2.5× more ^15^N by mass than 5th instar body samples (silk gland vs. bodies δ^15^N = 1.70‰ vs. 0.68‰). Within Site 2, 5th instar silk glands were 2.6× enriched in ^15^N relative to bodies (silk gland vs. bodies δ^15^N = 1.75‰ vs. −0.67‰), and were approximately 9.1% enriched in ^13^C (average body vs. silk gland δ^13^C = −23.34 vs. −21.33‰). Silk glands from 4th instar Site 1 larvae were similarly (9.2%) enriched in δ^13^C (average body vs. silk gland δ^13^C = −23.74 vs. −21.85‰) (Table 2, Figure 13).

#### 3.4.4. Adults

**Figure 14:**
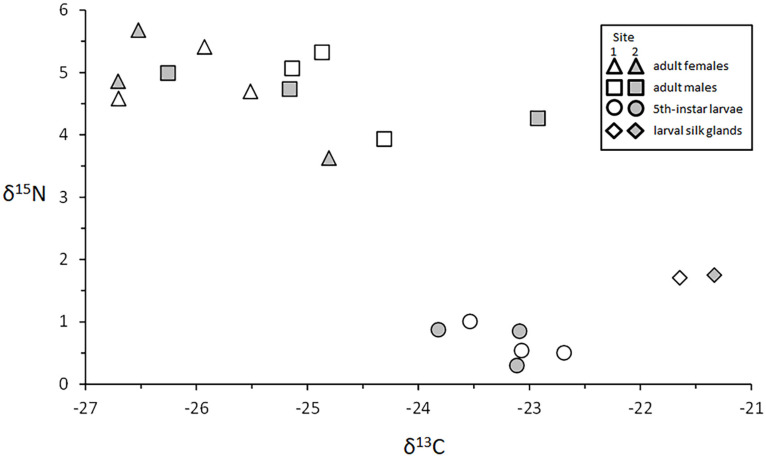
δ^13^C and δ^15^N data for male and female adults, 5th instar larvae, and 5th instar silk glands from Sites 1 and 2.

Adults from Sites 1 and 2 were significantly enriched in ^13^C and ^15^N relative to larvae (mean δ^13^C ± 1SE all adults vs. mean δ^13^C all larvae ± 1SE = −25.40 ± 0.32 vs. −23.23 ± 0.14, t(22) = −6.14, *p* < 0.00001). This strong pattern was also evident in contrasts between adults and 4th instars (t(16) = −4.37, *p* = 0.00048), and between adults and 5th instars (t(16) = −4.57, *p* = 0.00031). Within the sexes, δ^13^C and δ^15^N values were nearly identical between sites, as were between-sex δ^15^N values (Table 2). Females were, on average, significantly depleted in ^13^C relative to males (female mean ± 1 SE vs. male mean ± 1 SE = −26.03 ± 0.31 vs. −24.77 ± 0.45, t(22) = −2.28, *p* = 0.046). However, within sites, this effect was evident in Site 1 (t(10) −3.00, *p* = 0.040), but not in Site 2 (t(10) = −1.07, *p* = 0.34) (Figure 14).

## 4. Discussion

### 4.1. Life History

Rutherford and Mackay [67] conducted a detailed study of the life histories of five stream-dwelling *Hydropsyche* species within the range of *H. alternans*, and concluded that voltinism was “not immutable” in this group, with species showing variable numbers of annual cohorts in response to changes in food resource availability and water temperature. Our look at of the life history of *H. alternans* supports this observation, indicating that most, but perhaps not all, of the population in 2020 belonged to a cohort that overwintered in the 4th or 5th instars, pupated in the spring, and enclosed as adults between mid-June and late-August. Eggs laid on our samplers and black light sampling indicated that oviposition started around the summer solstice and peaked in the first weeks of July. Eggs on the samplers included hatched and hatching larvae at the time of collection (Figure 4)**.** The samplers were in the water for approximately 28 days; thus, the maximum time for substrate location by females, oviposition, and embryonic development was less than 4 weeks. This is consistent with past reports of Hydropsychidae embryonic development times, which range from 13–22 days under field conditions [65,67,68] and 8–11 days in the lab [69].

Larvae grew rapidly through August, with many achieving the 4th instar and some their 5th by the middle of the month. From September through October, much of population had molted to the 5th and final instar, and were presumably in the process of accumulating energy reserves for overwintering and reproduction. It seems likely that larvae that overwinter in the penultimate 4th instar complete their development, while the bulk of the population is pupating in the spring, but winter growth and development cannot be ruled out.

While most of the population in 2020 appeared to show univoltine development, we also observed evidence of a possible smaller second cohort that overwintered as 2nd or 3rd instars. Our inability to capture second cohort larvae in our early season samplers could have been due in part to instar-specific dispersal behaviors. To end up on one of our plate-style samplers, larvae had to have either been there as eggs or happened upon the sampler while repositioning by wandering or drifting. *Hydropsyche* larvae in streams disperse by drifting downstream throughout development, exhibiting the typical aquatic insect behavior of increasing drifting behavior just after dusk and right before dawn, to limit detection by visual feeding fish [70]. Local environmental factors, population density, season, and life stage are also known to affect *Hydropsyche* drifting behavior [70,71,72]. Larvae also commonly reposition by wandering in search of new retreat locations [73]. Flows in our sites fluctuate in strength, direction, and duration, so larval drifting could conceivably allow for movement in multiple directions whenever there are waves. Longshore currents that form during and after strong surf events could also allow for riverine-like “downstream” drifting along the coast. We assume that *H. alternans* and other surf zone hydropsychid larvae also commonly crawl to relocate, perhaps along with drifting, but we have yet to observe these behaviors.

So how did the nearly two thousand *H. alternans* larvae that we collected end up on our samplers? Due to the presence of egg masses and hatching larvae, we know that some of the 1st instar larvae were eclosed on our samplers. Beyond this, we know that some of the larvae, particularly later instars, must have dispersed to our samplers, because four weeks would have been insufficient time for them to have achieved the 4th or 5th instar. The instar distribution for all of our samplers combined was 14, 24, 10, 34, and 18% for instars 1–5, respectively. The relatively large percentage of 2nd instars (24%) could reflect a dispersal away from the natal substrate and/or 1st instars remaining on the natal substrate and molting to the second instar before the samples were retrieved. Instar 3 was clearly under-represented, which could represent low mobility or low effectiveness of the samplers at attracting this life stage. Fourth instars were the most commonly collected larvae overall, indicating a relatively high mobility and attraction to the samplers. The much larger 5th instar larvae colonized the samplers at a lower rate than the 4th instars, but they were also quite mobile, which is consistent with past reports of high rates of drifting by late-instar hydropsychids [71,72]. Our results indicate that most of the *H. alternans* in our study area overwinter in the 5th instar, which would likely require mobility to avoid advancing and shifting ice.

Our understanding of the overwintering strategy of *H. alternans* in Lake Superior is limited to observations made before shore ice forms when larvae can be found near the shore, but in low densities compared to the autumn months. Key questions remain about what they do when ice accumulations form, whether they remain active under ice, and if their strategy or strategies differ from those of stream populations, which are also unknown. This rudimentary state of scientific understanding of *H. alternans* overwintering is generally consistent with that of the winter biology of most of the benthic organisms of the Great Lakes. This is particularly true of the winter life on the bottom of Lake Superior, which remains largely unexplored [74]. Based on the general observations made over the past two decades, the surf zone habitat in our research area is typically ice-free into January. During most years, shore ice accumulates by mid-winter and lake ice floes drift in, packing the nearshore habitat with ice. We suspect that *H. alternans* larvae overwinter beyond this ice accumulation zone, which would allow them to remain active or at least surrounded by liquid water throughout the winter. Stream-dwelling *Hydropsyche* species within the range of *H. alternans* cease net-spinning activity during the winter, but continue to feed on epilithic organisms [10]. If *H. alternans* larvae feed throughout the winter, the staple diet of epilithic algae could also be subsidized by pulses of offshore energy, which could be more available at this time of year, due to increased nearshore-offshore mixing [75,76] The abundance of some Great Lakes diatoms [77] and zooplankton species peaks during winter, which indicates that food may be plentiful for net-spinning caddisflies in retreat sites beyond the shore ice zone. For example, the fellow post-glacial colonist *Limnocalanus macrurus*, the dominant copepod in the lake and perhaps the lake’s most abundant animal, loads the pelagic realm with nauplii during winter reproduction [78]. Opportunities for learning more about the winter biology of *H. alternans* in Lake Superior present researchers with obvious technical challenges. Overcoming them may be the key to understanding their apparent divergence to post-glacial lacustrine conditions, and their continued dominance of rocky surf zone habitats in Lake Superior.

### 4.2. Adult Characteristics

Larval crawling and drifting accounts for movement within habitat patches; for example, newly open retreat locations in the form of our plate samplers, but population persistence in isolated habitat patches such as exposed bedrock slabs along the shores of Lake Superior likely requires aerial dispersal and habitat recognition from above. The 2020 flight period of *H. alternans* at our sites extended over eight weeks. Adult hydropsychids typically live for one to two weeks on the wing [79,80], indicating that late-instar larval development, pupation, adult emergence, mating, and oviposition occur simultaneously throughout mid-summer. If *H. alternans* has adult dispersal capabilities similar to those of its congeners *H. hageni* and *H. phalerata* in Lake St. Clair, which lies between Lakes Huron and Erie, most females oviposit close to their larval habitat, but long-distance dispersal over 5 km occurs within a subset of the population [81]. Sode and Wiberg-Larsen [82] reported that forested stream populations of *H. pellucidula* and *H. siltalai* also maintained low levels of long-distance dispersal. These observations indicate that *Hydropsyche* females can survey the landscape and underwater habitats from the air to identify specific habitat features required for larval development. Because females attach eggs directly to the substrates on which larval development begins [79], the cues that they use to identify specific habitat types must differ among species with differing habitat requirements. Modern distribution records suggest that the ancestral habitat search parameters for *H. alternans* include silt-free, rocky substrates under clear waters in forested watersheds within a dynamic glacial landscape. The extension of the basal lotic habitat search parameters to include large lake littoral zones allowed this lineage and a few others within the family to exploit ecological opportunities available to attached, suspension-feeding animals in the vast expanses of large lake shorelines that have periodically formed and disappeared within their range over the past 800,000 years (the Pleistocene glacial–interglacial period).

The final step in this possible habitat divergence may have occurred when female *H. alternans* began to crawl or dive into proglacial lakes to oviposit. Most female Hydropsychidae oviposit underwater, and many species are known to utilize widened, paddle-shaped segments on their middle legs to swim to substrates (Figure 7B) [60,61]. Using an underwater light trap, Fremling [83] determined that *Hydropsyche orris* and *Cheumatopsyche campyla* females could swim down to 3.6 m in depth in the Mississippi River at a site where the channel is approximately 1 km wide, and *Potamyia flava* females swam down to an astounding 6 m. Oviposition was most concentrated at approximately 1 m, but the light trap illustrates the remarkable swimming abilities of these big river hydropsychids. We did not witness *H. alternans* oviposition in Lake Superior, but the presence of their eggs on our substrates 8 m from shore in 1.5 m deep water strongly suggests that the females can recognize lake bottom substrates from above, and are capable of deep oviposition. Deutsch [60] proposed that significant sexual dimorphism in mesothoracic leg shape, estimated using the length–width ratios of the first tarsal segments of the middle legs of females and males, was indicative of deep-diving and swimming behaviors. Specifically, females known to swim had ratios of between 2.9–4.7, compared to conspecific male ratios of 7.0–10.3. Our sample of the *H. alternans* population at Sites 1 and 2 had an average female ratio of 3.54 and a male ratio of 6.99, which indicates that female *H. alternans* legs are adapted for swimming. Barton and Hynes [1] suggested that large lake hydropsychids may not dive into surf zone waters, writing that “… this technique would seem hazardous, at best, on wave swept shores.” Although not incontrovertible, our observations indicate that *H. alternans* females in Lake Superior dive and swim to oviposit. Along with the aerial recognition of lake bottom substrates, diving, swimming, and deep oviposition behaviors may have been key adaptations allowing habitat expansion to glacial lake surf zones.

### 4.3. Gut Content Analyses

Larval hydropsychids are capable of feeding in multiple ways, a fact often overlooked in the trophic ecology literature. Capturing particles with nets is undoubtedly the primary mode of feeding when there is an abundance of high-quality food items present, but grazing algae, especially diatoms from nearby substrates and hunting for small invertebrates, are also common behaviors [9,10,15,84], particularly during winter in north-temperate waters when net-spinning is abandoned for general grazing [10]. Meiofaunal organisms that live in lake periphyton assemblages are known to disperse using wave energy to drift to new substrates [18,85]. They can also to be displaced by powerful waves that sweep them into suspension [86]. Moving in these ways, diatoms as well as meiofaunal animals such as benthic crustaceans would seem to be vulnerable to capture by hydropsychid nets. It is thus difficult to assign a feeding mode, i.e., filtering, grazing, collecting, or hunting, to hydropsychid gut content observations. Our gut content analyses, which were conducted on 4th and 5th instar larvae collected in October, offer a snapshot of the diet of large larvae at a time late in the growing season when benthic algal production has slowed from early summer peaks and strong surf is frequently experienced.

Diatoms were found in nearly all of the guts examined and thus appear to be a staple component in of the diets of surf zone net spinners. Fuller and Mackay [10] determined that diatoms contributed more than any other food type to the growth of all instars of three *Hydropsyche* species, *H. betteni*, *H. slossonae*, and *H. sparna*, which are hypothesized to be closely related to *H. alternans* [49]. Our algal identifications were too general to allow us to assess the relative importance of any particular diatom species in the *H. alternans* diet, but these closely related *Hydropsyche* species are known to discriminate among diatom species, including the active avoidance of *Melosira* sp., a chain-forming planktonic group with heavily silicified frustules [10]. If exhibited by *H. alternans*, this type of selectivity, particularly if it also includes benthic diatoms, could influence algal community structure and govern energy flows between benthic and pelagic food web components. Like most aspects of nearshore primary production and herbivory in Lake Superior, the details of these important trophic interactions await discovery. The research opportunities are particularly promising for collaborative endeavors of phycologists and entomologists.

Being selective feeders, hydropsychids are also known to avoid ingesting mineral particles [10], yet most of the larvae examined from Sites 1 and 2 had ingested relatively large amounts of sediment (Figure 8). It seems unlikely that they would consume this material without nutritional gain, and thus, we suspect that it was consumed incidentally while grazing on epilithic organisms such as epipsammic diatoms and other organisms that are bound along with fine sediments in the mucilaginous epilithon. This material, which has a pinkish color, has also been observed in the guts of other surf zone insects, including *Antocha* crane flies (Limoniidae) [3], which scrape epilithic algae from positions within silken tubes that are often built alongside surf zone hydropsychid retreats [1] (Figure 8).

#### 4.3.1. Carnivory

Approximately half of the larvae from Sites 3 and 4 had arthropod remains in their guts (Table 1, Figure 12). This is most likely an underestimate of actual carnivory, because we were only able to positively identify arthropod exoskeleton fragments, leaving unrecorded any soft-bodied forms or other invertebrates that could have been consumed and macerated beyond recognition. A review of the literature indicates that most, it not all larval hydropsychids consume animal prey when available, although differences in the degree of carnivory among larval instars and among species have been widely observed [68,84,87,88,89,90]. Carnivory starts early for some, including close relative of *H. alternans* and *H. slossonae*, which have been shown to engage in extensive sibling cannibalism shortly after larval eclosion [65]. Although we did not observe cannibalistic behavior, Figure 4B shows a situation where newly eclosed *H. alternans* larvae remained for some time within the egg mass with their seemingly defenseless younger siblings.

Diet studies conducted on larval hydropsychids have generally shown increases in carnivory in later instars [10,84]. Our results were consistent with this pattern. Specifically, 5th instar larvae were significantly enriched in ^15^N relative to 3rd instars (Table 2 and Table 3, Figure 10), indicating that carnivory increased as the larvae grew. It is also possible that the dramatic size increase that occurs during the final larval molt (Figure 3) allows 5th instar larvae to capture a broader range of prey, including smaller carnivores, which could result in ^15^N enrichment without necessarily increasing carnivorous tendencies. There is much left to learn about predatory behavior by *H. alternans*. Our results suggest that larvae employ a mixed feeding strategy that combines suspension feeding, algal scraping, and frequent predation on smaller invertebrates that are either captured in or near retreats, or encountered during repositioning maneuvers.

#### 4.3.2. Mycophagy

Although we do not know the taxonomic identity or identities of the fungal hyphae consumed by Site 3 larvae, the likely source of this material was the eroding beach dune face found just up-current from where we collected, as can be seen in Figure 9. Figure 9 also shows a side-by side comparison of fungal remains from the gut of a Site 3 larva and hyphae collected from the dune face near the hydropsychid habitat (Figure 9). This photographic evidence seems to confirm our suspicion about the source of the material. The ecological role of this fungus within the dune is likely mycorrhizal, and it may have been associated with vigorous patches of beach grass (*Ammophila breviligulata*) that line much of the shoreline at Site 3. The deep roots of *A. breviligulata* typically support extensive growths of mycorrhizae [91], which link dune plants and greatly extend their water and nutrient uptake capacities. These symbioses result in large fluxes of photosynthetically derived sugars into the hyphal network that permeates and stabilizes beach sands [92]. This sugar is converted by fungi into lipids, then transported through the hyphal web to be metabolized for energy, or stored in germ tubes and sporangia [93]. In the fall and early winter, large surf runs up unfrozen beaches, drawing large amounts of terrestrial organic matter into the surf zone (Figure 9). This is when most *H. alternans* larvae are in their final instar and accumulating resources for pupation and reproduction in the spring. Dune hyphae and other surf-delivered terrestrial subsidies could represent important sources of high-quality food for *H. alternans* larvae facing a long winter of predictably harsh environmental conditions.

The specimens that we used for gut content analyses were collected in October, when surf-producing storms are frequent. We do not have the wave height data for these sites, but past conditions can be assessed to a general level with wind speed and direction data from a NOAA Buoy, 60 km north-northeast of the research area (Stannard Rock, Station STDM4). Conditions in the two days leading up to the Site 4 collection date (6 October 2017) featured sustained strong west winds in the 50 km per hour range. All of our sites are sheltered from west winds (Figure 2), so on the collection day at Site 4, when conditions must be calm to collect from shore, wave action had been minimal for days. The conditions prior to the Site 3 collection were also stormy, but the wind direction produced strong surf. Over the course of 15 October 2017, the day before our collection date, gale-force, east-northeast to north winds battered the fully exposed coast. During a six-hour period in the middle of the day, winds averaged 76 km per hour, which is strong enough to cause significant dune erosion. If we are correct in our assessment of the source of the hyphae consumed by Site 3 larvae (Figure 9), then we expect that similar coastal terrestrial subsidies commonly fuel nearshore food webs following strong surf events. These beach and swash zone to surf zone connections may contribute substantially to nearshore energy flows, particularly in habitats downwind from thriving native dune vegetation communities.

### 4.4. Stable Isotope Analyses

#### 4.4.1. δ^13^C

If the hyphae that we found in larval guts from Site 3 were washed into the surf zone from an eroding dune face (Figure 9), then it is likely that larvae at Site 4, which is also partially fronted by sand beach with a vegetative community dominated by *Ammophila breviligulata*, also have periodic access to these and other materials that accumulate in swash zones and that become mixed into shoreline waters. Therefore, we did not expect that the significant difference in δ^13^C between larvae from Sites 3 and 4 was necessarily related to the presence of hyphae in the guts of the Site 3 larvae. We do suspect, however, that hyphae, which had recently been consumed by 76% of the *H. alternans* larvae at Site 3 (Table 1) are important sources of energy at these and other sites. Because the δ^13^C values for larvae that had hyphae in their guts spanned the range of values for the site (Figure 12), we predict that all larvae selectively consume hyphae when they can catch them from the flow.

The apparent source of the hyphal energy delivered to the surf zone in Site 3 was photosynthesis by beach vegetation, most likely by the deep and extensively rooted *Ammophila breviligulata*. Plants deliver energy to their mycorrhizal fungal partners in the form of hexose, the production of which preferentially utilizes ^13^C [94]. This ultimately produces isotopic separation between mycorrhiza symbionts, which ranges from 6 to 10‰ [95,96]. The hyphae consumed by *H. alternans* could also have been produced by saprotrophic fungi, which are generally enriched in ^13^C by 4‰ relative to their substrates [94]. Published average δ^13^C values for *Ammophila breviligulata* range from −28 to −27‰ [97,98]. The forest vegetation above the beach at Site 3 largely comprises red pines (*Pinus resinosa*). Similar to dune grass, red pine needle δ^13^C is approximately −28‰ [99], and the organic layer detritus of a similar red pine stand was reported by Mellilo et al. [100] to be −26.2‰. Therefore, assuming an approximate 6‰ enrichment and an approximate source δ^13^C of −27, the δ^13^C of some of the common fungal hyphae in this habitat would be approximately −21, or perhaps a few mil higher, as a result of hexose conversion to lipids, which would be expected to discriminate against ^13^C [96]. This is obviously a speculative estimate, but it is intriguingly close to the δ^13^C −21.3 average for Site 3 larvae. This larval average may simply reflect a balance between benthic and pelagic production, but it is plausible that larval δ^13^C at Site 3 was in part also a reflection of these terrestrial energy subsidies which were based on the amount of direct, up-current location during our most common surf-producing storms.

#### 4.4.2. δ^15^N

The reported average trophic position for lotic Hydropsychidae is 2.5 [17,23], and approximately half of the secondary production of southeastern US stream species is estimated to be derived from animal prey energy [87,88,89,90]. Therefore, our data, and those from a prior study [3], suggest that Lake Superior *H. alternans* occupies the typical lotic Hydropsychidae niche within the surf zone food webs. Figure 12 shows this niche to be roughly between herbivorous *Antocha* crane flies, which specialize on epilithic algae, and *Hydra*, which are suspension-feeding benthic carnivores. Our analysis of 3rd instar larvae from Site 3 indicated that they may have been less carnivorous than the much larger 4th and 5th instar larvae (Table 2, Figure 3 and Figure 10), which is consistent with past reports for stream-dwelling hydropsychids [84].

Gut content and δ^15^N analyses showed that the *H. alternans* diet and trophic position are typical of those of most well-known hydropsychids; i.e., they are opportunistic carnivores that become increasingly more carnivorous as they develop (Figure 10). It also seems likely that some of the apparent trophic separation that is observed between small and large larvae could have resulted from an increasing capacity to capture and kill larger prey as the larvae grow, including fellow predators. For example, the consumption of Tanypodinae midges (Diptera: Chironomidae) caught hunting among the lairs of 5th instar *H. alternans* would contribute substantially to the body’s ^15^N content. Future research that includes detailed taxonomic and seasonal analyses of *H. alternans* prey species is needed to allow for the identification of specific energetic connections linking surf zone net spinners to benthic and planktonic food webs via carnivory.

#### 4.4.3. Larval Silk Glands

Silk production has likely evolved multiple times within Insecta, allowing a myriad of lineages to address ecological challenges using various forms of this lightweight, strong, sticky, elastic, and waterproof wonder fiber [101]. For hydropsychid larvae, silk production is central to feeding and lodging strategies, and also allows them to rappel to safety and to find their way back home when dislodged by currents and crashing waves [102]. Along with the alimentary canal, the large and folded Z-shaped silk glands are the most obvious features of the internal anatomy of larval hydropsychids. We observed that the fullness of silk glands varied considerably among *H. alternans* larvae, with some being thin and apparently empty, while most were plump full of silk proteins and precursor molecules. As expected, the silk glands that we analyzed were enriched in ^13^C and ^15^N, relative to the body tissues (Table 2, Figure 13). This observation can be explained, at least in part, by the specific amino acid composition of the silk dope and the precursor materials stored within the glands. The major silk proteins of another *Hydropsyche* species (*H*. *angustipennis*) comprise roughly equal fractions of glycine, serine, and a combination of isoleucine, leucine, and valine [103]. In general, isotopic fractionation during nitrogen metabolism accounts for the progressive enrichment of ^15^N during trophic transfers, which is commonly assessed using δ^15^N bulk analyses. However, the relative amounts of ^15^N atoms that become sequestered into individual amino acids varies considerably. So too do the relative amounts of ^13^C in amino acid precursors. The major silk protein constituents glycine and serine exhibit strong positive δ^15^N and δ^13^C fractionation [104,105]. Isoleucine, leucine, and valine exhibit small positive fractionations of nitrogen, but no fractionation of carbon isotopes [104]. Therefore, compared to the whole body average, full silk glands have isotopic signatures that increase the estimated trophic position and shift the main energy source estimate toward benthic producer values, which is consistent with our results (Table 2, Figure 13). This suggests that a larva could, from the perspective of stable isotope analysis, undergo an apparent niche shift after expelling large amounts of silk. To avoid this potential source of error, which could have produced some of the variability observed in our analyses from Sites 3 and 4, silk gland fullness should be considered when selecting larvae for isotope analyses. This would be an easy addition to a protocol that already includes removing gut contents, which is recommended for carnivores and carnivorous omnivores like hydropsychids, to avoid prey remain contributions to analytical results [21].

#### 4.4.4. Adults

The physiological processes that occur during pupation produce major changes in isotopic values. Most of the energy and amino acids required for these biosynthesis activities is produced by the catabolism of larval proteins, while fat reserves from larval feeding are reserved for adult activities [106,107]. The significant ^15^N enrichment that we observed for adults relative to larvae (Figure 14) likely resulted from the excretion of ^15^N-depleted metabolic waste meconium produced during this period of extensive protein metabolism and voided at the end of pupation [108]. The depleted δ^13^C values of adults relative to larvae, and adult females relative to males, were likely due to the metabolism of ^13^C-enriched proteins catabolized during pupation, and to the relatively high concentration of ^13^C-depleted lipids stored for adult energy [106,107,109]. Although we do not know if *H. alternans* is an important component of the diets of tertiary consumers in Lake Superior, we suspect that emerging adults and ovipositing females diving and swimming with a sheen of air trapped within body setae could be easily spotted by fish, and thus may contribute substantially to the diets of native coastal fishes such as brook trout (*Salvelinus fontinalis*), lake trout (*Salvelinus namaycush*), and lake whitefish (*Coregonus clupeaformis*). We hope that the stable isotope values presented here for larval and adult *H. alternans* from Lake Superior will be useful in future attempts to clarify the roles that these common caddisflies and other native surf zone insects play in nearshore food webs.

## 5. Conclusions

The earliest record of net-spinning caddisflies in Lake Superior was the capture during the 1871 United States Lakes Survey of a single *Hydropsyche* larva in a dredge sample taken from 26 m deep water along the north shore, approximately 200 km northeast of our research area. The presence of *Hydropsyche* larvae in water so deep in Lake Superior is intriguing and begs for further exploration, but perhaps even more notable was a statement by Chief Zoologist Sidney I. Smith regarding the discovery. He reported that “the larvae, pupae, and sub-imago of the same or closely allied species were found in great abundance in stomachs of white-fish taken at Sault Sainte Marie” [110]. The predator–prey relationship that he described, quite possibly between the lake whitefish (*Coregonus clupeaformis*) and *Hydropsyche alternans*, illustrates the potential importance of net-spinning caddisflies and other native aquatic insects in the Lake Superior food web. We are still in the discovery phase of understanding most aspects of the ecology of most of these species. Barton and Hynes conducted the most comprehensive study on the nearshore insects of the Great Lakes to date. They considered this work to be “a preliminary step toward expanding our knowledge of the benthos of large lakes to include the unique habitat found in such bodies of water—namely the continuously wave-swept shores of the Great Lakes.” [1]. This study takes a preliminary step toward an understanding of one of the 29 native invertebrate species that they found along the rocky, surf-exposed shores of Lake Superior.

The high-latitude, periglacial conditions experienced by the westernmost documented population of *H. alternans* [49] (see Supplementary Materials S3: Periglacial Habitat) indicate that divergence to glacial lake conditions may have involved existing adaptations for survival just beyond the grip of the ice. These caddisflies live in a tributary to a small remnant of the once massive proglacial Lake Atna that existed for 50,000 years at the end of the Pleistocene [111]. Their environment may represent the ancestral conditions that selected for adaptations, allowing *H. alternans* to exploit isolated pockets of relatively productive riverine and wave-swept lacustrine habitats within the vast, harsh, and dynamic glacial landscape.

Earth’s rapidly changing climate has forced significant changes in environmental conditions in lakes globally [112]. Particularly pronounced effects of lake warming have occurred in Lake Superior, including increases in air–lake energy exchange, decreases in winter ice coverage [113,114], and the recent highly unusual occurrences of noxious cyanobacteria blooms in otherwise oligotrophic nearshore plankton assemblages during the warmest summers [115]. Many more life history studies like the one presented here for *H. alternans* are required to begin to understand the biological responses of native organisms of Lake Superior to these rapid, systemwide changes in this globally important freshwater resource.

Many of the historical collection records presented in Figure 1 are from watersheds that have been dramatically altered by human activities. These likely represent declining or extirpated populations, particularly in watersheds converted to large-scale agriculture [47], and in habitats that are densely populated by *Dreissena* mussels [30]. The rocky surf zone habitats in Lake Superior remain largely mussel-free at present, but the recent appearance of established juvenile and adult *Dreissena* in the Apostle Islands, 90 km from their likely source in the St. Louis River estuary [116] indicates that long-distance dispersal during the planktonic veliger stage is occurring along the south shore. A low-density *Dreissena* population exists on the iron ore loading dock in Marquette’s upper harbor, just down-current of our research area. It is not known whether this assemblage is a self-sustaining population or a group of displaced hitchhikers sustained by recruitment from established populations elsewhere in the Great Lakes. An established *Driessena* population in the Au Train river system, a popular canoeing and kayaking destination approximately 50 km down the coast from our research area, appears to pose an even greater threat to local surf zone insect communities (see Supplementary Materials S4: Tributary *Dreissena*). We fear that this and possibly other yet to be discovered tributary populations are poised to expand downstream to surf zone habitats. It is unclear whether environmental conditions in Lake Superior have thus far presented insurmountable challenges to invading mussels, or if *Dreissena* is slowly spreading [117,118]. What is certain is that anthropogenic changes to Earth’s atmosphere are affecting the limnological environment in Lake Superior [113], and that these changes will test the ancient adaptive strategies that have allowed *Hydropsyche alternans* to thrive in the last remaining stronghold of the surf zone insects of the Laurentian Great Lakes.

## Figures and Tables

**Table 1:** Gut contents from 39 *Hydropsyche alternans* larvae from two sites on the south-central shore of Lake Superior. Values are percentages of individuals that had recognizable remnants of the specified item in their guts. All guts also contained amorphous organic matter.

	Algae (Diatoms and Green Algae)	Animal (Arthropod Exoskeleton Fragments)	Fungi (Hyphae)	Sediment (Fine Particles)
**Site 3**	94	50	78	72
**Site 4**	95	48	0	57

**Table 2:** Sample sizes, means, standard errors, and data ranges from stable isotope analyses.

Sample Type	Site	n	Mean δ^13^C ± 1SE	Low δ^13^C	High δ^13^C	Mean δ^15^N ± 1SE	Low δ^15^N	High δ^15^N
** 3rd instar **	3	5	−22.95 ± 0.33	−23.94	−22.38	−1.26 ± 0.53	−2.90	0.29
** 4th instar **	3	4	−21.50 ± 0.40	−22.58	−20.77	−0.06 ± 0.23	−0.74	0.30
** 4th instar **	4	12	−22.48 ± 0.13	−23.37	−22.00	0.74 ± 0.13	−0.02	1.29
** 5th instar **	3	13	–21.28 ± 0.22	−22.48	−20.01	0.80 ± 0.23	−0.28	2.50
** 5th instar **	4	9	−22.83 ± 0.40	−25.82	−21.82	0.23 vs. 1.10	−0.06	1.70
** 4th instar **	1	3	−23.74 ± 0.12	−23.89	−23.51	−0.14 ± 0.17	−0.47	0.06
** 4th instar silk glands **	1	1 (n = 3)	−21.85			0.83		
** 4th instar **	2	3	−22.73 ± 0.25	−23.12	−22.28	0.08 ± 0.44	−0.77	0.68
** 4th instar silk glands **	2	1 (n = 3)	−20.38			1.25		
** 4th instar silk glands **	2	1 (n = 3)	−20.66			1.07		
** 5th instar **	1	3	−23.10 ± 0.25	−23.54	−22.69	0.68 ± 0.16	0.50	1.01
** 5th instar silk glands **	1	1 (n = 3)	−21.64			1.70		
** 5th instar **	2	3	−23.34 ± 0.24	−23.82	−23.11	0.67 ± 0.19	0.30	0.87
** 5th instar silk glands **	2	1 (n = 3)	−21.33			1.75		
** adult female **	1	3	−26.05 ± 0.35	−26.70	−25.51	4.89 ± 0.26	4.54	5.41
** adult female **	2	3	−26.01 ± 0.61	−26.52	−24.80	4.72 ± 0.60	3.63	5.68
** adult male **	1	3	−24.77 ± 0.24	−25.14	−24.31	4.77 ± 0.43	3.93	5.32
** adult male **	2	3	−24.78 ± 0.98	−26.26	−22.92	4.66 ± 0.21	4.26	4.99

**Table 3:** Tukey–Kramer post-hoc contrasts, means, and *p*-values for analyses of δ^13^C and δ^15^N data for 3rd, 4th, and 5th instar larvae from Site 3. *p*-values indicating statistically significant differences are highlighted in bold text.

Contrast	*p*-Value δ^13^C	*p*-Value δ^15^N
Instar 3 × Instar 4	** 0.0271 **	0.1235
Instar 3 × Instar 5	** 0.0027 **	** 0.0007 **
Instar 4 × Instar 5	0.9482	0.2251

**Table 4:** Tukey–Kramer post-hoc contrasts, means, and *p*-values for analyses of δ^13^C and δ^15^N values from 4th and 5th instar larvae collected from Sites 3 and 4. Significant *p*-values are highlighted in bold text.

Contrast	*p*-Value δ^13^C	*p*-Value δ^15^N
Site 3 Instar 4 × Site 3 Instar 5	0.9683	0.0958
Site 3 Instar 4 × Site 4 Instar 4	0.1817	0.1384
Site 3 Instar 4 × Site 4 Instar 5	** 0.0486 **	** 0.0189 **
Site 3 Instar 5 × Site 4 Instar 4	** 0.0047 **	0.9956
Site 3 Instar 5 × Site 4 Instar 5	** 0.0006 **	0.6686
Site 4 Instar 4 × Site 4 Instar 5	0.7615	0.5513

## Data Availability

Voucher specimens are housed in the Northern Michigan University Insect Collection.

## Supplementary Materials

The following supporting information can be downloaded at: https://www.mdpi.com/article/10.3390/insects13070659/s1, Supplementary Materials S1: Taxonomic History [119,120,121,122,123,124,125,126,127,128,129,130,131,132,133], Supplementary Materials S2: Detailed Site Descriptions [134,135], Supplementary Materials S3: Periglacial population [49,111,136], Supplementary Materials S4: Tributary *Dreissena.*
