# Supplementary material for: An Ecological Profile of Hydropsyche alternans (Trichoptera: Hydropsychidae) in Lake Superior, the Last Stronghold of a Once-Dominant Great Lakes Surf Zone Caddisfly
Source: Insects. 2022 Jul 21;13(7):659. doi: 10.3390/insects13070659 (PMC9325119; doi:10.3390/insects13070659)

Supplementary Materials S1: Taxonomic History

The long, strange history of *Hydropsyche alternans* (Walker) taxonomy includes multiple species descriptions, genus designations, and a long-standing lack of consensus about which of its past names is valid. The species that we presently call *Hydropsyche alternans* was first described from specimens collected during the 1830s from Martin's Falls (51˚37'N, 86˚23'W) on the Albany River, about 420 kilometers upstream from its mouth on James Bay (Figure 1)**.** George Barnston, an amateur naturalist who worked at a fur trading post on the Albany, collected these and thousands of other specimens, including hundreds of species new to science, which he delivered to the British Museum where prolific taxonomist Francis Walker gave it the name *Philopotamus alternans* [119]. In 1914, the species was described again as new to science by American entomologist Nathan Banks in 1914 who named it the *recurvata* variant of *Hydropsyche slossonae* due to the distinctively curved spur on the aedeagus. The type specimen for this designation was an adult male collected by Canadian entomologist E.M. Walker early in June on Go Home Bay, Lake Huron**,** a University of Toronto Biological Station approximately 2100 km southeast of its original type locality [120]. It was once again described as new in 1934 by Betten who collected the species he named *Hydropsyche codona* in Ogdensburg, New York on the St. Lawrence River about 100 km from its source at the Lake Ontario outflow of the Great Lakes [121]. This name didn't stand long as Ross, in 1938, determined that the distinctive male genitalia from Betten's description of *H. codona* were identical to those of *H. recurvata,* which he also elevated to species status [122]. To complete the confusing first century of *Hydropsyche alternans* taxonomy, Betten [121] proposed the name *Hydropsyche alternans* as a synonym for several species that he declared were the same as those originally described by Walker as *Philopotamus alternans*. However, the illustrations he used to distinguish this species from other *Hydropsyche* species, which incidentally, he noted were "sometimes common on rocks and piers along the shore of Lake Michigan" (121:186), look to actually be *H. bifida*, which was described by Banks in 1905 [123]. Nimmo [124] determined this to be the case, and that *H. bifida* Banks was also the true identity the species described as *Hydropsyche alternans* by Vorhies in 1909 [125]. Nimmo [124] can also be credited for determining that Walker's original species name *alternans* had priority over Banks' *recurvata* and as a result, the common surf zone net spinner had its name: *Hydropsyche alternans*. However, just as *H. retrocurva* in early literature refers to *H. alternans*, over the past 50 years it was also briefly known as *Symphitopsyche recurvata* [126] and for a longer time as *Ceratopsyche alternans* [e.g., 3, 127-130] as researchers adopted proposals to split *Hydropsyche* into genera representing widely accepted species-groups within the genus. At present, a more conservative "lumping" approach appears to have gained wide acceptance [e.g., 131-133] and all former *Ceratopsyche* species have been returned to *Hydropsyche,* at least until more is known about this ancient lineage's representatives in understudied regions [133].

Supplementary Materials S2: Detailed Site Descriptions

Shoreline habitat and vegetation type designations follow the Michigan Natural Features Inventory-Michigan State University Extension Service Natural Community Classification system [134].

Site 1 lies just north of the mouth of the Little Garlic River, which often floods the site with tannin brown river water**.** This site is the most sheltered from direct exposure to the strongest surf-producing winds received in the research area, the vast majority of which blow from the north and northwest. Occasionally storm force north to northwest winds produce strong surf in Site 1 as waves wrap around Thoney Point and refract into the river mouth area. South and southeast wind swell affect this site more than the other sites, but the relatively short fetch limits the potential size and strength of northward moving waves along this stretch of coast. As can be seen in the satellite photo on Figure 2, the Little Garlic River water often flows in a northerly direction, creating a clockwise eddy flow of tannin-stained, dark-brown river water that circulates through the mouth area as it mixes with lake water. The lakeshore here is not sandstone pavement as is the case in the other sites. At this site, fluvial sand and gravel deposits are overlain with a narrow band of boulder substrates that line the coast. The substrates and vegetation are similar to sandstone cobble lakeshore habitat with river mouth and sand-and-gravel-beach directly to the south. Hydropsychid habitat is limited to a 10-30 m. wide strip of boulders and rounded cobbles that meet a large fluvial sand deposit.

Site 2 is a small east-facing sandstone point. Strong north winds produce surf that generally wraps around our 60-meter long research site, creating riverine-like longshore current flows. Similar, but typically weaker, longshore currents flowing north are a frequent occurrence during summer months when southerly and southeasterly winds are common. The substrates and vegetation are characteristic of sandstone-bedrock lakeshore habitat. Stable sandstone crack and inter-rock crevice habitat within the surf zone is very abundant and extends north and south of the site.

Site 3 is a State of Michigan Natural Area called Little Presque Isle, a name that reflects the fact that the sandstone "neck" of this granitic headland has weathered enough to form a nearshore island and approximately 150 meters of sandstone and boulder habitat between the island and the mainland. Strong north winds have the interesting effect of producing waves that break directly on the sandstone from the north and also from the south as waves refract around the headland island**.** This site features a combination of river mouth, sandstone-bedrock lakeshore, granite-bedrock lakeshore, and sand-and-gravel-beach habitats. High-quality hydropsychid habitat is abundant within the zone connecting the island and shore and along the coast to the south. The partial embayment to the north features a shifting river mouth (Harlow Creek) and a stretch of sandy beach that captures large volumes of organic debris in the swash zone which gets washed into the surf zone during strong surf events.

Site 4 is narrow strip of sandstone cobble habitat fringing the west side of an ancient volcanic plug associated with continental rifting 1 billion years ago [135]. This island-like land mass, which is

broadly connected to the shoreline, is a City of Marquette park called Presque Isle Park. The bay on the west side of the volcanic "island" (Middle Bay) is well known for its surf. Strong north and northwest winds channel waves through a 10-meter deep trough in the middle of the bay. The points and islands on the west and northwest side of the bay direct outward current flows along the prime hydropsychid habitat. This site is sheltered from southerly winds and most easterly winds although gale force east winds produce waves that flow into the bay. High quality hydropsychid habitat is abundant along the eastern shore of the bay. Shoreline substrates and vegetation include sandstone bedrock lakeshore, granite-bedrock lakeshore, and sand-and-gravel-beach habitats.

Supplementary Materials S3: Periglacial Population

The western-most *H. alternans* collection record from Schefter and Wiggins [49] is from a stream 120 km from the Gulf of Alaska. Alaska's Mendeltna Creek is a tributary to Tazlina Lake which lies at the terminus of the Tazlina glacier. The waters downstream from these caddisflies feature high concentrations of fine, "glacial flour" sediments, presumably making them uninhabitable for caddisflies [136]. Tazlina Lake itself is a remnant of glacial Lake Atna, a massive proglacial lake that existed for 50,000 years at the end of the Pleistocene [111]. The *H. alternans* population at this site occupies a small patch of suitable habitat, with the potential to disperse by flight to exploit pockets of relatively productive riverine and lacustrine habitats within a dynamic glacial landscape.

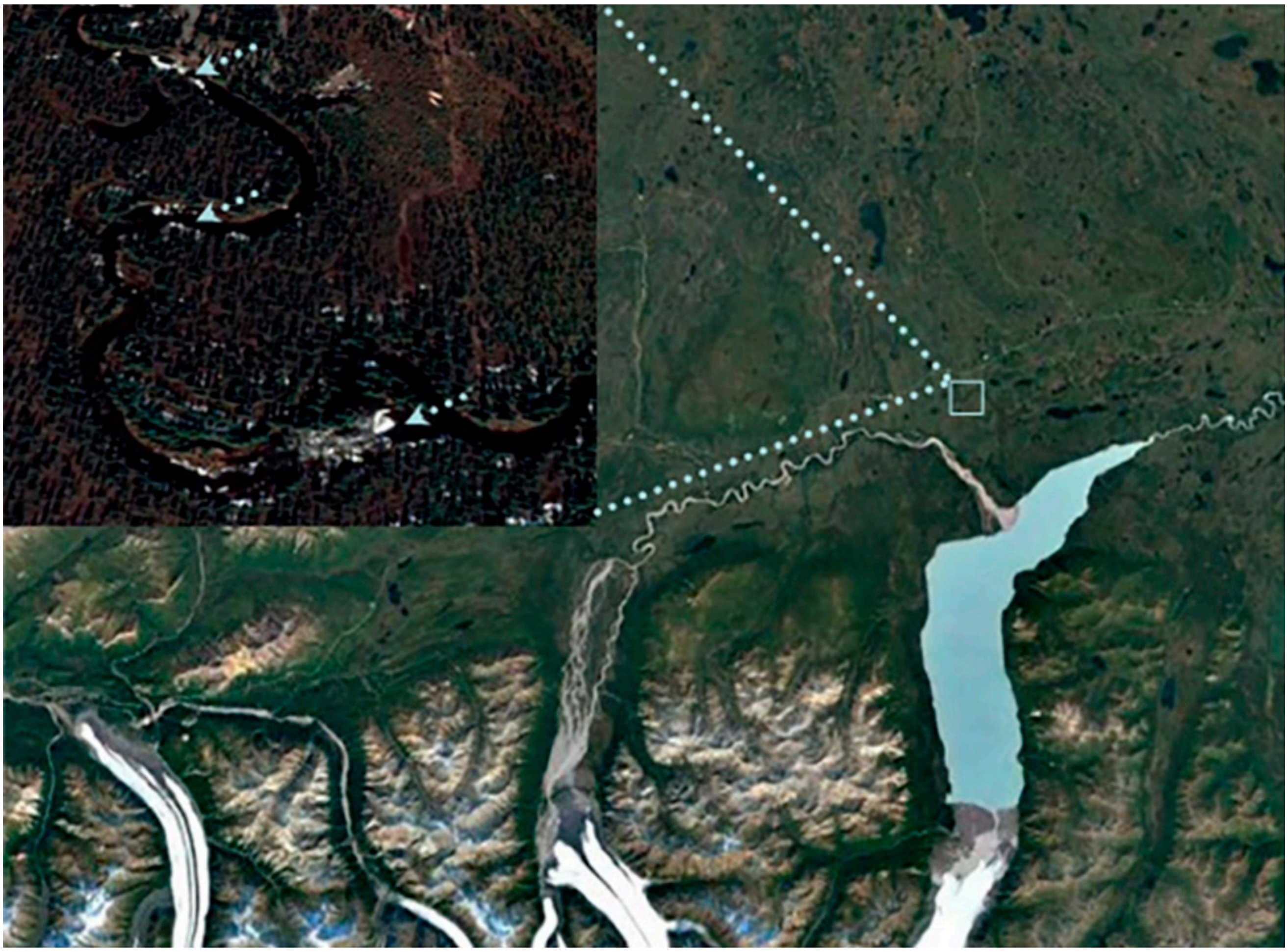

Supplementary Materials S4: Tributary *Dreissena*.

Photo documentation of *Dreissena polymorpha* and *Cipangopaludina chinensis* in the Au Train River system. A – Adult *Dreissena* attached to a native unionid mussel 2.4 km. upstream from Lake Superior; B – *Dreissena* and invasive mystery snails (*Cipangopaludina chinensis*) in an upstream reservoir. These photographs were taken in August 2017.

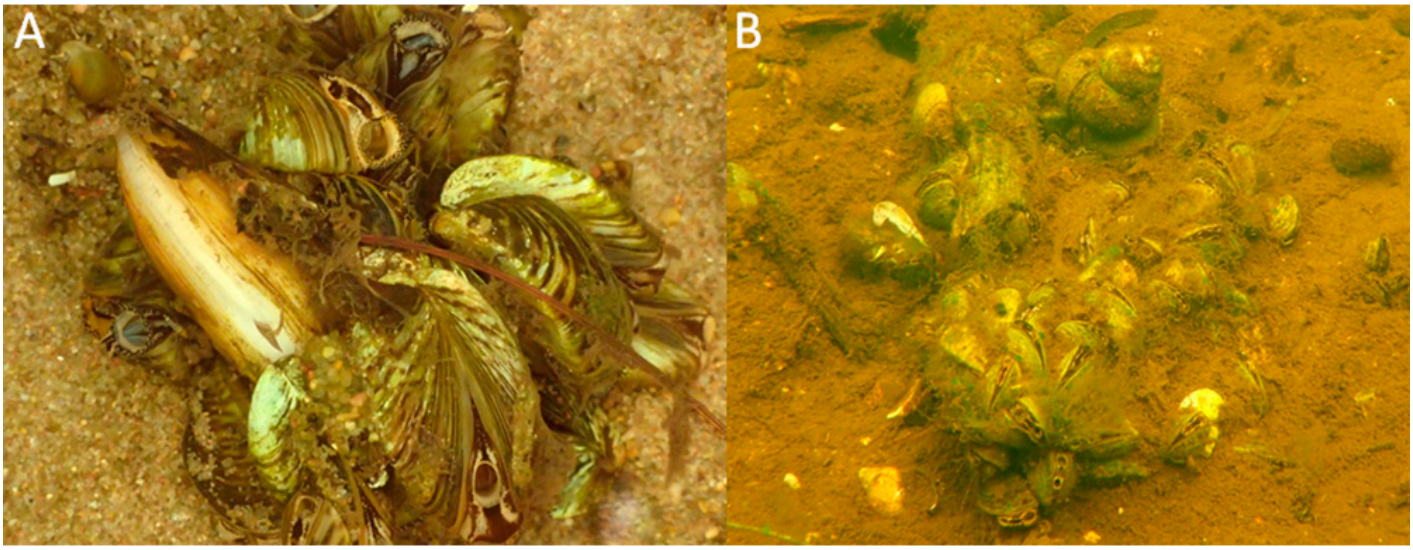

Supplement: Supplementary file 1 [file insects-13-00659-s001.zip › insects-1509398-supplementary.pdf]
